# The Most Relevant Factors Affecting the Perioperative Death Rate in Patients with Acute Coronary Syndrome and COVID-19, Based on Annual Follow-Up in the ORPKI Registry

**DOI:** 10.3390/biomedicines9121813

**Published:** 2021-12-02

**Authors:** Karol Kaziród-Wolski, Janusz Sielski, Jacek Sidło, Rafał Januszek, Zbigniew Siudak

**Affiliations:** 1 Collegium Medicum, Jan Kochanowski University in Kielce, 25-369 Kielce, Poland; kk-wolski@wp.eu (K.K.-W.); jaceksidlo@wp.pl (J.S.); zbigniew.siudak@gmail.com (Z.S.); 2 Department of Cardiology and Cardiovascular Interventions, University Hospital, 2 Jakubowskiego Street, 30-688 Krakow, Poland; jaanraf@interia.pl; 3 Second Department of Cardiology, Faculty of Medicine, Institute of Cardiology, Medical College, Jagiellonian University, 30-688 Krakow, Poland

**Keywords:** COVID-19, acute coronary syndrome, STEMI

## Abstract

Background: The COVID-19 pandemic is significantly affecting the functioning of the entire healthcare system. The disease itself may be associated with thromboembolic complications. The purpose of this study is to compare patients with acute coronary syndrome (ACS) and patients with ACS who were diagnosed with COVID-19 in terms of their clinical profile, management, treatment complications, and prognosis. Methods: We analyzed 47,940 cases of patients treated for ACS in 2020, including 44,952 patients (93.8%) who were not diagnosed with COVID-19 and 2988 patients (6.2%) who tested positive for COVID-19. Results: Patients with COVID-19 were significantly more likely to experience out-of-hospital sudden cardiac arrest (7.9 vs. 1.1%; *p* < 0.0001) and be transported directly to a catheterization laboratory (21.3% vs. 8.1%; *p* < 0.0001). Mortality was significantly higher in this group (0.9% vs. 0.4%; *p* < 0.0001). The risk of perioperative death was increased by age over 65 years, use of glycoprotein IIb/IIIa inhibitors (GPI IIb/IIIa), femoral access, critical left main stem coronary artery (LMCA) vascular lesions, ST elevation myocardial infarction (STEMI), and no-reflow phenomenon. Conclusions: Despite the pandemic, patients with COVID-19 were treated equally to healthy patients. Efficient organization of the healthcare system allowed the prompt transportation of patients to catheterization laboratories. The study group was characterized by a worse prognosis that was affected by multiple factors.

## 1. Introduction

The first cases of infection with the new coronavirus were recognized in China in 2019. The beginning of the epidemic occurred in Wuhan province [1]. Within a few months, the epidemic, then a pandemic, spread around the whole world. To date, 229,858,719 cases of the disease have been recorded worldwide (including 4,713,543 deaths). In Poland, as of September 2021, 2,901,674 cases have been recorded (including 75,551 deaths) [2]. Despite countermeasures coordinated on a large scale by the World Health Organization (WHO), there has been an unprecedented spread of the pandemic.

Since the most life-threatening changes during the disease occur in the respiratory system, the International Committee on Taxonomy of Viruses named this virus severe acute respiratory syndrome coronavirus 2 (SARS-CoV-2) [3]. On 11 February 2020, the WHO officially used the name COVID-19 in the context of the disease caused by the SARS-CoV-2 virus [4]. The disease COVID-19 was initially classified into four degrees of severity: mild, moderate, severe, and critical [5]. Most viral infections are asymptomatic, but in some cases, viral infection causes severe pneumonia, progressing to respiratory failure [5]. Infections are diagnosed by reference genetic testing, such as reverse transcription–polymerase chain reaction (RT-PCR). Lesions in the respiratory system are diagnosed based on emergency chest computed tomography (CT) scans [5,6]. Although COVID-19 is a new disease, there is a lot of information about the influence of the infection on cardiovascular diseases, including acute coronary syndrome (ACS). The viral infection promotes ruptures of unstable atherosclerotic plaque, and the cytokine storm increases local inflammation [7]. Another aspect that affects the management of ACS patients infected with SARS-CoV-2 is the risk of nasopharyngeal infection in other uninfected patients as well as medical staff [7].

The prevailing pandemic has changed the functioning of both individual citizens and societies in general [8]. Before the pandemic, a patient diagnosed with ACS was appropriately qualified and sent to a reference center for reperfusion treatment [9]. Such management was performed based on European and national guidelines [9,10]. The pandemic changed the algorithm of patient management, especially in COVID-19-positive or suspected patients [11]. This management was broadly implemented to protect medical staff and other patients from becoming infected [11,12].

The aim of this study was to track and determine the differences in management between patients with ACS and those who were additionally diagnosed with COVID-19. We aimed to identify the most significant factors that contributed to increased perioperative mortality in the catheterization laboratory.

## 2. Materials and Methods

The National Registry of Invasive Cardiology Procedures (Ogólnopolski Rejestr Procedur Kardiologii Inwazyjnej, ORPKI) was established in 2004 on the initiative of cardiologists from Krakow. Data on the management of ACS have been collected since 2004. The electronic ORPKI database started working in January 2014. The Registry was created by the Association of Cardiovascular Interventions of the Polish Cardiac Society and is currently coordinated by the Jagiellonian University Medical College in Krakow. The centers of invasive cardiology in Poland which are registered in ORKPI report to the electronic database of the Registry. Currently, 161 catheterization laboratories from Poland are included in the registry.

So far, the management of patients with ACS has followed the guidelines of the Polish Cardiac Society. The outbreak of the epidemic, and then the pandemic that also affected Europe and Poland, introduced the need for different management of patients diagnosed with COVID-19. Such management is consistent with the principles of invasive treatment among COVID-19-positive (+) patients in accordance with the principles of self-protection and the protection of uninfected patients staying in the same hospital.

The study group consisted of patients undergoing invasive treatment for ACS in 2020. On 8 March 2020, the first COVID-19 infection was detected in Poland. In that year, there was a sharp increase in the number of COVID-19 cases. The peak of infections in Poland occurred on 24 November 2020, reaching 44,035 cases. The maximum daily number of deaths due to COVID-19 in Poland was recorded on 25 November 2020 (a total of 505 patients). This was the “second wave” of infections [13]. The group of patients collected for analysis, namely those who qualified for treatment of ACS and were registered in ORPKI, included 47,940 patients. This group consisted of 44,592 patients without COVID-19 infection (93.8%) and 2988 patients who were diagnosed with COVID-19 (6.2%). A positive result was determined by the result of an antigen test performed in the ambulance or at the destination hospital. Due to the need to adhere to time standards, PCR test results were not waited for. Patients with suspected COVID-19 (as recommended for triage by the National Institute of Public Health and the Ministry of Health) were treated as potentially COVID-19(+). The COVID-19 diagnosis was always available before any interventional procedure (angiography or percutaneous coronary intervention) and was recorded in the ORPKI online database. Swabs for molecular RT-PCR were always obtained before the procedure.

We performed a pooled analysis of comorbidities in patients with ACS, as well as in those with ACS and COVID-19. Moreover, any predisposing factors and drugs used in ACS and COVID-19 treatment were analyzed. Two groups of patients were compared: patients with ACS but without confirmed infection (COVID-19(−)) and patients with ACS in addition to confirmed infection (COVID-19(+)). We performed a univariate regression analysis of the clinical, prehospital, and pharmacological factors to the endpoint of perioperative death. The patients who qualified for invasive treatment signed informed consent forms in accordance with the 1964 Helsinki Declaration recommendations. Since we used anonymous data from the ORPKI database, the study did not require the approval of the Bioethics Committee.

### Statistical Analysis

Quantitative variables are expressed as means (SD) and medians (interquartile range). Categorical variables are presented as numbers and percentages. The normality of the data distribution was checked by the Kolmogorov–Smirnov test. The χ^2^ test was used to evaluate statistical significance in the two-way tables. The Mann–Whitney test for variables with an abnormal distribution was used to assess the intragroup differences. One-dimensional and multiple logistic regression models were used to estimate odds ratios with 95% confidence intervals and *p*-values. Forest charts were used to present statistically significant odds ratios. The evaluation of multivariate logistic regression models was performed using ROC analysis; the ROC curves are shown graphically. A *p*-value of less than 0.05 was considered significant. The statistical analysis was performed using Med-Calc Statistical Software, version 20.0 (MedCalc Software, Ostend, Belgium) [12].

## 3. Results

In total, 47,940 patients treated for ACS throughout 2020 were analyzed. There were 44,952 COVID-19(+) patients and 2988 COVID-19(−) patients. Clinical characteristics revealed that patients with COVID-19 were younger, with less frequent episodes of previous myocardial infarction, percutaneous coronary intervention (PCI) or coronary aortic bypass grafting (CABG), and arterial hypertension. Patients with COVID-19 were more often transported directly to the catheterization lab and more often experienced out-of-hospital cardiac arrest (Table 1).

Patients with COVID-19 more often received saturating doses of acetylsalicylic acid, unfractionated heparin (UFH), low molecular weight heparin (LMWH), strong P2Y12 inhibitors, and GPI IIb/IIIa inhibitors. During coronarography in patients with COVID-19, femoral access was more frequent. Fractional flow reserve (FFR) was performed less frequently, and the dose of radiation and contrast was higher in patients with COVID-19 (Table 2).

Patients with COVID-19 were characterized by less frequent right coronary artery (RCA) stenosis and more frequent left anterior descending artery (LAD) stenosis, with a lower number of stents implanted. Patients with COVID-19 had a higher percentage of periprocedural deaths as well as a more frequent occurrence of any periprocedural complications. There were no significant differences between COVID-19(+) and (−) patients in the incidence of stroke, dissection, injection site bleeding, allergic reactions, and the no-reflow phenomenon (Table 3).

One-way logistic regression showed the effect of age > 65 years and ST elevation myocardial infarction (STEMI) on perioperative mortality in the COVID-19(+) group, similar to the COVID-19(−) group (Table 4).

The one-way logistic regression analysis showed the effect of GP IIb/IIIa inhibitors and femoral access during coronarography on periprocedural mortality (Table 5). Among the anatomical factors and complications, critical left main coronary artery (LMCA) stenosis, and the no-reflow syndrome had a significant impact (Table 6).

**Figure 1:**
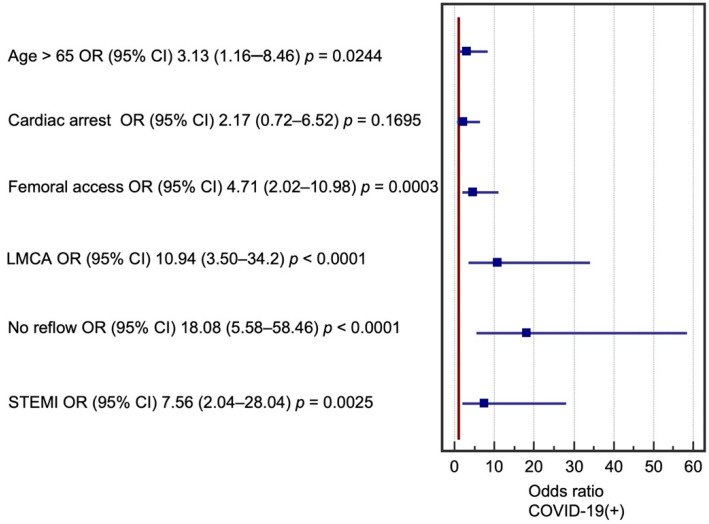
Factors affecting periprocedural mortality in COVID-19(+) patients. CI—confidence interval, LMCA—left main coronary artery, OR—odds ratio, STEMI—ST elevation myocardial infarction.

**Figure 2:**
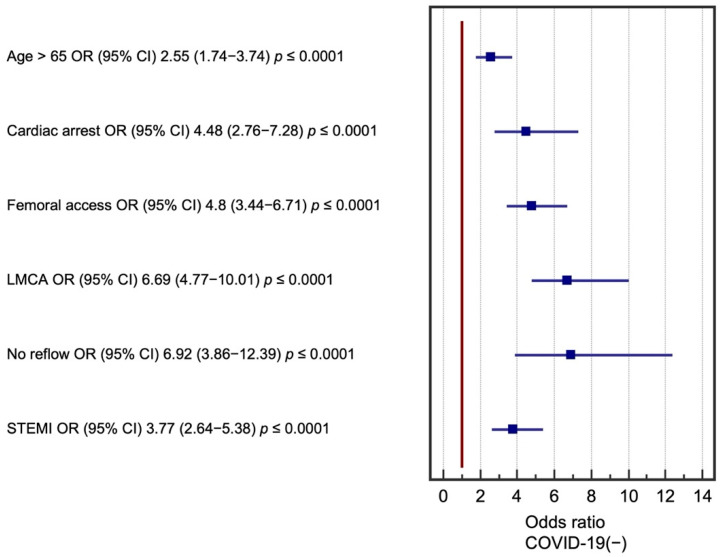
Factors affecting periprocedural mortality in COVID-19(−) patients. CI—confidence interval, LMCA—left main coronary artery, OR—odds ratio, STEMI—ST elevation myocardial infarction.

**Figure 3:**
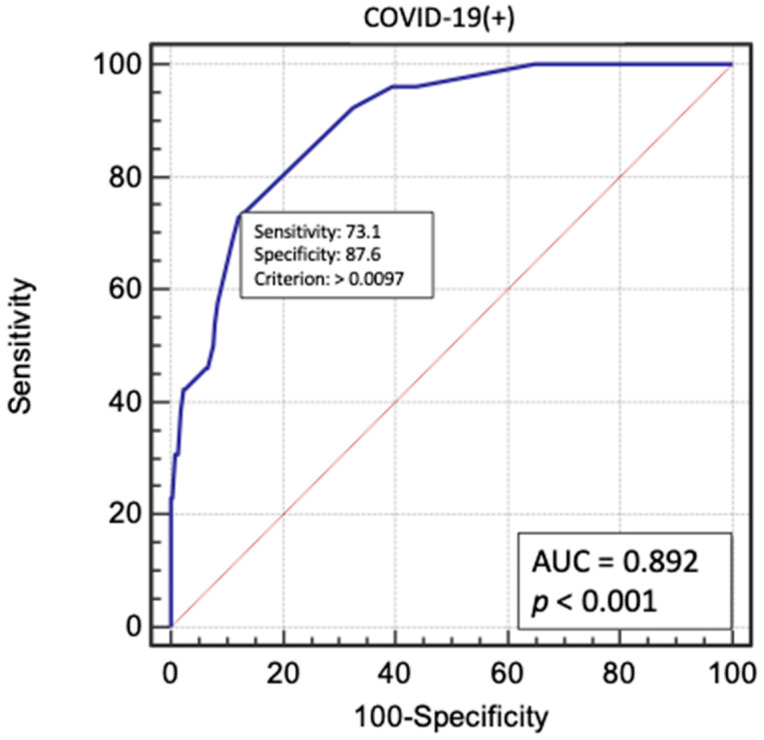
Receiver operating characteristic (ROC) curve for the multivariate logistic regression model in COVID-19(+) patients. AUC—area under curve.

**Figure 4:**
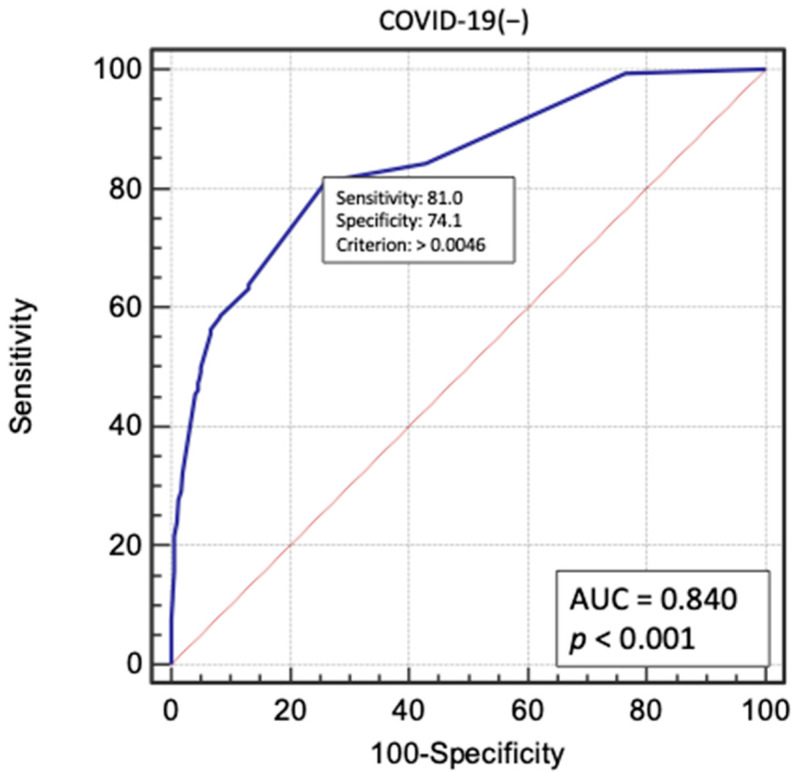
Receiver operating characteristic (ROC) curve for multivariate logistic regression model in COVID-19(−) patient. AUC—area under curve.

Multivariate logistic regression analysis was performed for significant factors in the COVID-19(+) group. The following factors had the greatest impact on perioperative death in the two groups: age over 65 years, use of GCI IIb/IIIa inhibitors in therapy, femoral access during angiography, critical LMCA lesions, occurrence of STEMI, and no reflow in the perioperative period. Cardiac arrest before coronary angiography was found to be a significant prognostic factor, but only in the group of patients who were not diagnosed with COVID-19. These results are presented in Figure 1 and Figure 2. Multivariate logistic regression models were evaluated using receiver operating characteristic (ROC) curves (Figure 3 and Figure 4).

## 4. Discussion

The COVID-19 pandemic has changed the functioning of societies in many important ways. Healthcare operations have also changed significantly, not only in the treatment of COVID-19 and related diseases, but also in the treatment of other conditions. One of the conditions where treatment and management has changed significantly during the pandemic is ACS. Siudak et al. compared the 2-month periods from March to May in 2019 and 2020 in terms of the number of treated ACS cases. The number of STEMI cases decreased in 2020 by 36%, non-ST-segment elevation myocardial infarction (NSTEMI) cases by 39%, and unstable angina cases by 58% compared with 2019 [12]. The total number of coronary angiographies decreased significantly from 172,521 in 2019 to 130,662 in 2020. The number of percutaneous coronary interventions decreased from 101,716 in 2019 to 82,349 in 2020 [14]. Similar trends in invasive cardiology procedures were observed to have been affected by the pandemic across Europe. De Rosa et al. described a significant reduction in the number of patients admitted to hospitals due to ACS in 2020: reductions of 26.5% for STEMI patients and of 65.1% for NSTEMI patients [15]. Similarly, Papafaklis et al. found a significant reduction in admissions during comparable periods of 28.4% for ACS, 24.5% for STEMI, 26.5% for NSTEMI, and 36.5% for unstable angina [16].

Besides the analysis of the number of procedures, it is very important to observe and analyze the individual perioperative factors affecting prognosis during ACS treatment of COVID-19(+) and COVID-19(−) patients. One of the most important perioperative factors is the time between the onset of chest pain and the start of revascularization (balloon inflation or angiogram). In the comparative analysis we performed, more COVID-19(+) patients than COVID-19(−) patients were brought to catheterization laboratories in under 12 hours. This demonstrates the efficient organization of the emergency medical and invasive cardiology services. More COVID-19(−) patients had a revascularization onset time exceeding 48 hours. It can be speculated that the psychological impact of the patient played a decisive role in this delay. Similarly, Matsushita et al. compared a group of 106 COVID-19(+) patients treated in 2020 with a group of 174 patients treated in 2019, and found a reduction in the time between first medical contact and stent implantation in STEMI patients [17]. A similar comparative study of patients from the ORPKI database was conducted by Siudak et al. In the data from 2 months of 2020, there were no differences in the time from first medical contact to balloon inflation, onset of pain to first medical contact, onset of pain to balloon inflation, or first medical contact to balloon inflation [12]. Koutsoukis et al. performed a comparative analysis on a relatively small group of patients with ACS (*n* = 121, COVID-19(+) *n* = 9, COVID-19(−) *n* = 112) and found no differences in the time to revascularization between the groups [18]. The psychological aspect of the late arrival of COVID-19(−) patients for invasive diagnostics is known from previous publications and can be observed in the form of fewer invasive cardiology procedures being performed [19,20].

An important issue in the analysis of COVID-19(+) patients undergoing invasive treatment for ACS is the problem of transporting patients directly to catheterization labs. In our analysis of 2988 COVID-19(+) patients, we found that more of them used direct transportation. This was undoubtedly due to the good organization of the emergency medical services, the separation and availability of “COVID-19 ambulances,” the separation of hospitals dedicated to treating COVID-19 patients, and COVID-19 cardiology units in hospitals providing normal healthcare during the pandemic [21].

A very important factor that significantly affects the perioperative death rate is sudden cardiac arrest before admission to the hospital. In our study, cardiac arrest affected 1.5% of the patients overall. In this group, a significantly higher percentage of patients were COVID-19(+) than COVID-19(−) (7.9% vs. 1.1%, respectively). The reason for this remains unclear. There are probably many factors involved. The COVID-19(+) patients were in a more serious condition, with symptoms of heart and respiratory failure, and advanced infection. No comparison of perioperative death in ACS for COVID-19(+) and COVID-19(−) patients was found in the available publications.

Sielski et al. and Tokarek et al. analyzed the aspect of perioperative death among patients in the ORPKI database and found significant correlations between sudden pre-hospital cardiac arrest in ACS and perioperative death from ACS [22,23].

In the analysis of COVID-19(+) and COVID-19(−) patients with ACS undergoing invasive cardiology procedures, two other factors were noteworthy: a significantly higher number of infected patients who received GPI IIb/IIIa inhibitors during the procedure and the notably lower number of stents implanted during the procedure. The former can be explained by the developed thrombotic process in COVID-19(+) patients. This problem has been described in the literature [24,25].

The developed thromboembolic process in COVID-19(+) was confirmed by the significant effect of the no-reflow phenomenon on perioperative death that we observed. This corresponds with the study of Güler et al., who investigated factors affecting the course of STEMI in COVID-19(+) and COVID-19(−) patients. In an analysis of 62 patients with STEMI, the no-reflow phenomenon was found to have a significant effect on the course of STEMI and coronary artery bypass graft [26]. Our study confirms the highly significant effect of no-reflow on the course and perioperative death rate in ACS and COVID-19(+) patients.

The discussion referred to the problem of gender differences in the ACS. Differences in the specificity and management of ACS in men and women have been described for many years. Many studies have confirmed many important differences. In a large review, Pagidipati et al. confirmed worse short- and long-term prognosis, especially in the group of young women [27]. On the other hand, Davis et al., in a study of 3237 patients with ACS in women and men, confirmed a higher incidence of diabetes and arterial hypertension. On the other hand, mortality was lower and did not change over the years. In women, there was less qualification for invasive treatment. At the same time, rehospitalizations were more frequent [28]. COVID-19 is a severe systemic disease that has spread worldwide in a pandemic. Precisely how it affects the course of ACS in men and women will be the subject of many studies in the future.

In the COVID-19 pandemic, more is needed than an analysis of the number of hospitalizations for ACS. It seems much more important to identify significant differences in the impact of relevant perioperative factors observed and recorded during the management of patients with ACS. Analyzing significant perioperative factors in large medical registries such as ORKPI serves to do this.

### Limitations of the Study

The limitation of this study is that data for the ORPKI database were collected by catheterization labs in Poland before invasive cardiology procedures. Since qualification for the procedure was immediate, it was not possible to wait for biochemical results. The patients were not qualified for procedure based on biomarkers released into the blood. The procedures were applied following the current guidelines. Thus, in our research, based on data from the ORPKI database, we do not have data on biomarkers, and we do not refer to them.

## 5. Conclusions

1. During the pandemic, COVID-19(+) patients diagnosed with ACS underwent invasive cardiology procedures on par with COVID-19(−) patients, although the overall number of procedures has decreased, according to previous studies.
2. The organization of the emergency medical rescue allowed COVID-19(+) patients with ACS to reach invasive diagnostic centers in a shorter time than non-infected patients.
3. The development of thromboembolic processes in COVID-19 patients results in an unfavorable course of coronary angioplasty (no-reflow phenomenon) and promotes higher perioperative mortality.
4. COVID-19(+) patients arrive at the cardiology centers quickly, have significantly developed thromboembolisms, and must be treated with appropriate drugs. They have a worse prognosis and higher perioperative mortality.

## Figures and Tables

**Table 1:** Characteristics of clinical and prehospital management factors.

Variable	Total	COVID(−)	COVID(+)	*p*-Value
Clinical Factors				
*n*	47,940	44,952 (93.8)	2988 (6.2)	<0.0001
Sex (male)	30,874 (64.6)	28,814 (64.3)	2060 (69.4)	<0.0001
Age, median (Q1–Q3)	67 (60–74)	67 (60–74)	66 (60–74)	0.0043
Age (>65 years)	27,257 (56.9)	25,708 (57.2)	1549 (51.8)	<0.0001
Diabetes (*n*, %)	10,740 (22.4)	10,097 (22.5)	643 (21.5)	0.2316
Previous stroke (*n*, %)	1474 (3.1)	1368 (3.0)	106 (3.5)	0.1221
Previous MI (*n*, %)	10,713 (22.3)	10,241 (22.8)	472 (15.8)	<0.0001
Previous PCI (*n*, %)	12,326 (25.7)	11,867 (26.4)	459 (15.4)	<0.0001
Previous CABG (*n*, %)	2407 (5.0)	2303 (5.1)	104 (3.5)	0.0001
Smoking (*n*, %)	10,587 (22.1)	9850 (21.9)	737 (24.7)	0.0004
Psoriasis (*n*, %)	303 (0.6)	287 (0.6)	16 (0.5)	0.5081
Hypertension (*n*, %)	32,249 (67.3)	30,474 (67.8)	1775 (59.5)	<0.0001
Kidney disease (*n*, %)	2728 (5.7)	2552 (5.7)	176 (5.9)	0.5583
COPD (*n*, %)	1702 (3.6)	1580 (3.5)	122 (4.1)	0.1041
Prehospital Management				
Acute coronary syndrome (*n*, %)				0.3390
STEMI	11,746 (24.5%)	10,312 (22.9%)	1434 (48.0%)	
NSTEMI	13,600 (28.4%)	12,682 (28.2%)	918 (30.7%)	
Unstable angina	22,594 (47.1%)	21,958 (48.8%)	636 (21.3%)	
Time from pain to first contact (*n*, %)				0.3390
<12 h	16,064 (78.4)	14,555 (78.4)	1509 (79.9)	
12–48 h	4318 (21.3)	3946 (21.3)	372 (19.7)	
>48 h	74 (0.4)	65 (0.4)	9 (0.5)	
Time from pain to inflation or angiogram (*n*, %)				<0.0001
<12 h	12,156 (58.4)	10,898 (57.7)	1258 (65.2)	
12–48 h	6024 (28.9)	5548 (29.4)	476 (24.7)	
>48 h	2648 (12.7)	2454 (13.0)	194 (7.3)	
Time from first contact to inflation or angiogram (*n*, %)				<0.0001
<12 h	16,460 (79.6)	14,697 (78.6)	1763 (79.6)	
12–48 h	3340 (16.1)	3145 (16.8)	3340 (16.1)	
>48 h	884 (4.3)	845 (4.5)	884 (4.3)	
Direct transport to catheterization lab (*n*, %)	4259 (8.9)	3622 (8.1)	637 (21.3)	<0.0001
Cardiac arrest at baseline	726 (1.5)	489 (1.1)	237 (7.9)	<0.0001

CABG—coronary aortic bypass grafting; COPD—chronic obstructive pulmonary disease; MI—myocardial infarction; OHCA—out of hospital cardiac arrest; PCI—percutaneous coronary intervention, NSTEMI—non-ST elevation myocardial infarction, STEMI—ST elevation myocardial infarction.

**Table 2:** Characteristics of pharmacological and periprocedural factors.

Variable	Total	COVID(−)	COVID(+)	*p*-Value
Pharmacological Factors				
ASA (*n*, %)	17,607 (36.7)	15,877 (35.3)	1730 (57.9)	<0.0001
UFH (*n*, %)	11,116 (23.2)	9994 (22.2)	1122 (37.6)	<0.0001
LMWH (*n*, %)	1396 (2.9)	1246 (2.8)	150 (5.0)	<0.0001
P2Y12 inhibitor (*n*, %)	16,066	14,926 (92.9)	1140 (7.1)	<0.0001
clopidogrel	9086 (56.6)	8577 (57.5)	509 (44.6)	
prasugrel, and ticagrelor	6980 (43.4)	6349 (42.5)	631 (55.4)	
Thrombolysis (*n*, %) (47,940)	11 (0.02)	11 (0.02)	0	0.3925
GPI IIb/IIIa during angiogram (21,146) (*n*, %)	4373 (20.7)	3976 (19.8)	397 (36.4)	<0.0001
Bivalirudin (*n*, %)	4 (0.008)	4 (0.009)	0	0.6061
Periprocedural Factors				
IVUS (*n*, %)	492 (1.0)	468 (1.0)	24 (0.8)	0.2115
OCT (*n*, %)	35 (0.07)	32 (0.07)	3 (0.1)	0.5670
Vascular access (*n*, %)				<0.0001
Radial	42,088 (88.8)	39,558 (89.0)	2530 (85.7)	
Femoral	5307 (11.2)	4884 (11.0)	423 (14.3)	
FFR (*n*, %)	1376 (2.9)	1341 (3.0)	35 (1.2)	<0.0001
Total amount of contrast used during procedure, mL (IQR)	120 (80–170)	120 (70–170)	130 (100–180)	<0.0001
Total radiation dose, mGy (IQR)	404 (198–771)	398 (191–760)	491 (255–869)	<0.0001

ASA—acetylsalicylic acid; FFR—fractional flow reserve; GPI IIb/IIIa—IIb/IIIa glycoprotein inhibitor; IQR—interquartile range; IVUS—intravascular ultrasonography; LMWH—low molecular weight heparin; OCT—optical coherence tomography; PCI—percutaneous coronary intervention; TIMI—thrombolysis in myocardial infarction; UFH—unfractionated heparin.

**Table 3:** Characteristics of coronary arteries anatomy, implanted stents, and complications during procedures.

Variable	Total	COVID(−)	COVID(+)	*p*-Value
Critical Stenosis of Coronary Artery and Implanted Stents				
RCA (*n*, %)	9941 (31.9)	9280 (32.1)	661 (28.8)	0.0011
LMCA (*n*, %)	1034 (3.3)	952 (3.3)	82 (3.6)	0.4702
LAD (*n*, %)	12,254 (39.3)	11,178 (38.7)	1076 (47.0)	<0.0001
SvG (*n*, %)	398 (1.3)	371 (1.3)	27 (1.2)	0.6610
LIMA/RIMA (*n*, %)	58 (0.2)	57 (0.2)	1 (0.04)	0.1002
Bifurcation (*n*, %)	3352 (10.8)	3109 (10.8)	243 (10.6)	0.8061
DES (*n*, %)	27,325 (87.7)	25,451 (88.1)	1874 (81.9)	<0.0001
BVS (*n*, %)	29 (0.09)	29 (0.1)	0	0.1290
BMS (*n*, %)	51 (0.2)	49 (0.2)	2 (0.09)	0.3473
Number of implanted stents (*n*, %)				<0.0001
0	3737 (12.1)	3351 (11.6)	416 (18.2)	
1	22,314 (71.6)	20,752 (71.9)	1562 (68.2)	
≥2	5086 (16.3)	4772 (16.5)	314 (13.7)	
Complications During Procedures				
Total (*n*, %)	350 (0.7)	309 (0.7)	41 (1.4)	<0.001
Death (*n*, %)	199 (0.4)	172 (0.4)	27 (0.9)	<0.0001
Stroke (*n*, %)	8 (0.02)	8 (0.02)	0	0.4658
Dissection (*n*, %)	34 (0.07)	30 (0.07)	4 (0.1)	0.1822
Bleeding at the puncture site (*n*, %)	24 (0.05)	21 (0.05)	3 (0.1)	0.2020
Allergic reaction (*n*, %)	9 (0.02)	8 (0.02)	1 (0.03)	0.5449
No reflow (*n*, %)	342 (0.9)	317 (0.9)	25 (1.3)	0.1098

BMS—bare metal stent; BVS—bioresorbable vascular scaffold; DEB—drug-eluting balloon; DES—drug-eluting stent; LAD—left anterior descending; LMCA—left main coronary artery; LIMA/RIMA—left internal mammary artery/right internal mammary artery; RCA—right coronary artery; SvG—saphenous vein graft.

**Table 4:** Clinical and prehospital management factors affecting periprocedural mortality.

Variable	COVID(−)		COVID(+)	
	OR (95% CI)	*p*-Value	OR (95% CI)	*p*-Value
Sex (male)	0.69 (0.51–0.94)	0.0176	1.55 (0.62–3.85)	0.3443
Age (>65 years)	2.48 (1.74–3.53)	<0.0001	3.28 (1.32–8.16)	0.0105
Diabetes	1.26 (0.90–1.77)	0.1785	0.45 (0.14–1.51)	0.1976
Previous stroke	3.30 (1.96–5.53)	<0.0001	2.20 (0.51–9.40)	0.2883
Previous MI	0.84 (0.57–1.22)	0.3456	1.53 (0.61–3.81)	0.3613
Previous PCI	0.42 (0.28–0.67)	0.0002	0.68(0.21–2.29)	0.5407
Previous CABG	1.39 (0.77–2.51)	0.2715	*	
Smoking	0.72 (0.48–1.08)	0.1101	0.53 (0.18–1.53)	0.2407
Psoriasis	2.78 (0.88–8.76)	0.0806	*	
Hypertension	0.74 (0.55–1.01)	0.0587	0.63 (0.30–1.35)	0.2355
Kidney disease	2.20 (1.38–3.51)	0.0010	2.82 (0.97–8.25)	0.0582
COPD	1.15 (0.78–2.98)	0.2237	0.90 (1.12–6.11)	0.9203
STEMI (vs. NSTEMI and UA)	6.5 (4.74–8.9)	<0.0001	8.8 (2.64–29.29)	0.0004
Time from pain to first contact, (<12 h, 12–48 h, >48 h)	0.93 (0.61–1.42)	0.7250	1.27 (0.42–3.79)	0.6743
Time from pain to inflation or angiogram, (<12 h, 12–48 h, >48 h)	0.69 (0.52–0.91)	0.0085	1.12 (0.58–2.17)	0.7416
Time from first contact to inflation or angiogram, (<12 h, 12–48 h, >48 h)	0.41 (0.24–0.70)	0.0012	0.43 (0.07–2.78)	0.3719
Direct transfer to catheterization lab	3.96 (2.81–5.59)	<0.0001	1.06 (0.42–2.63)	0.9083
Cardiac arrest at baseline	17.86 (11.73–27.21)	<0.0001	3.38 (1.35–8.45)	0.0093

* There was no death during the procedure for positive values of the variable, and the authors did not include this variable in the regression model. CABG—coronary aortic bypass grafting; COPD—chronic obstructive pulmonary disease; MI—myocardial infarction; NSTEMI—non-ST elevation myocardial infarction; OHCA—out of hospital cardiac arrest; OR—odds ratio; PCI—percutaneous coronary intervention, STEMI—ST elevation myocardial infarction; UA—unstable angina.

**Table 5:** Pharmacological and periprocedural factors affecting periprocedural mortality.

Variable	COVID(−)		COVID(+)	
	OR (95% CI)	*p*-Value	OR (95% CI)	*p*-Value
ASA	2.75 (2.02–3.73)	<0.0001	1.06 (0.49–2.29)	0.8856
UFH	2.41 (1.77–3.27)	<0.0001	1.15 (0.53–2.48)	0.7311
LMWH	1.50 (0.70–3.20)	0.3021	0.73 (0.10–5.39)	0.7540
Prasugrel and ticagrelor(vs. clopidogrel)	1.58 (0.84–2.96)	0.1561	1.08 (0.24–4.83)	0.9238
Thrombolysis	*		*	
GPI Iib/IIIa during angiogram	12.22 (6.17–24.21)	<0.0001	12.47 (1.53–31.53)	0.0184
Bivalirudin	*		*	
IVUS	1.12 (0.28–4.52)	0.8649	*	
OCT	*		*	
Vascular access (femoral)	9.16 (6.76–12.44)	<0.0001	7.71 (3.58–16.60)	<0.0001
FFR	*		*	
Total amount of contrast, ml	1.005 (1.004–1.006)	<0.0001	1.004 (0.999–1.008)	0.0637
Total radiation dose, mGy	1.001 (1.000–1.001)	<0.0001	1.0002 (0.9996–1.0007)	0.2860

* There was no death during the procedure for positive values of the variable, and the authors did not include this variable in the regression model. ASA—acetylsalicylic acid; FFR—fractional flow reserve; GPI IIb/IIIa—IIb/IIIa glycoprotein inhibitor; IVUS—intravascular ultrasonography; LMWH—low molecular weight heparin; OCT—optical coherence tomography; OR—odds ratio; PCI—percutaneous coronary intervention; TIMI—thrombolysis in myocardial infarction; UFH–unfractionated heparin.

**Table 6:** Coronary anatomy, implanted stents, and complications during procedures affecting periprocedural mortality.

	COVID(−)		COVID(+)	
Variable	OR (95% CI)	*p*-Value	OR (95% CI)	*p*-Value
RCA	0.43 (0.28–0.65)	0.0001	0.91 (0.38–2.18)	0.8331
LMCA	9.73 (6.75–14.04)	<0.0001	8.59 (3.36–22.01)	<0.0001
LAD	1.87 (1.37–2.55)	0.0001	1.32 (0.61–2.87)	0.4800
SvG	0.97 (0.24–3.94)	0.9616	*	
LIMA/RIMA	3.21 (0.44–23.32)	0.2493	*	
Bifurcation	1.11 (0.69–1.79)	0.6713	0.70 (0.16–2.98)	0.6280
DES	0.24 (0.17–0.33)	<0.0001	0.60 (0.25–1.43)	0.2464
BVS	*		*	
BMS	3.73 (0.51–27.20)	0.1937	*	
Number of implanted stents	0.46 (0.34–0.61)	<0.0001	0.90 (0.45–1.79)	0.7676
0				
1				
≥2				
Complications				
Stroke	*		*	
Dissection	9.02 (1.22–66.62)	0.0308	*	
Bleeding at the puncture site	*		*	
Allergic reaction	*		*	
No reflow (*n*, %)	11.50 (6.77–19.53)	<0.0001	19.03 (6.70–54.06)	<0.0001

* There was no death during the procedure for positive values of the variable, and the authors did not include this variable in the regression model. BMS—bare metal stent; BVS—bioresorbable vascular scaffold; DEB—drug-eluting balloon; DES—drug-eluting stent; LAD—left anterior descending; LMCA—left main coronary artery; LIMA/RIMA—left internal mammary artery/right internal mammary artery; OR—odds ratio; RCA—right coronary artery; SvG—saphenous vein graft.

## Data Availability

The datasets generated during and/or analyzed during the current study are available from the corresponding author on reasonable request.
